# Tissue specific analysis reveals a differential organization and regulation of both ethylene biosynthesis and E8 during climacteric ripening of tomato

**DOI:** 10.1186/1471-2229-14-11

**Published:** 2014-01-08

**Authors:** Bram Van de Poel, Nick Vandenzavel, Cindy Smet, Toon Nicolay, Inge Bulens, Ifigeneia Mellidou, Sandy Vandoninck, Maarten LATM Hertog, Rita Derua, Stijn Spaepen, Jos Vanderleyden, Etienne Waelkens, Maurice P De Proft, Bart M Nicolai, Annemie H Geeraerd

**Affiliations:** 1 Division of Mechatronics, Biostatistics and Sensors (MeBioS), Department of Biosystems (BIOSYST), KU Leuven, Willem de Croylaan 42, bus 2428, 3001 Leuven, Belgium; 2 Department of Microbial and Molecular Systems, Center of Microbial and Plant Genetics, KU Leuven, Kasteelpark Arenberg 20, bus 2460, 3001 Leuven, Belgium; 3 Department of Cellular and Molecular Medicine, KU Leuven, Herestraat 49, 3000 Leuven, Belgium; 4 Division of Crop Biotechnics, Department of Biosystems, KU Leuven, Willem de Croylaan 42, 3001 Leuven, Belgium; 5 Flanders Centre of Postharvest Technology (VCBT), Willem de Croylaan 42, 3001 Leuven, Belgium; 6 Department of Cell Biology and Molecular Genetics, University of Maryland, Bioscience Research Bldg 413, College Park, MD 20742, USA; 7 Division of Chemical and Biochemical Process Technology and Control Section, Department of Chemical Engineering, KU Leuven, Willem de Croylaan 46, 3001 Leuven, Belgium

**Keywords:** *Solanum lycopersicum*, Tomato, Ethylene biosynthesis, Tissues, Pericarp, Septa, Columella, Placenta, Seeds, Locular gel, E8

## Abstract

**Background:**

*Solanum lycopersicum* or tomato is extensively studied with respect to the ethylene metabolism during climacteric ripening, focusing almost exclusively on fruit pericarp. In this work the ethylene biosynthesis pathway was examined in all major tomato fruit tissues: pericarp, septa, columella, placenta, locular gel and seeds. The tissue specific ethylene production rate was measured throughout fruit development, climacteric ripening and postharvest storage. All ethylene intermediate metabolites (1-aminocyclopropane-1-carboxylic acid (ACC), malonyl-ACC (MACC) and *S*-adenosyl-L-methionine (SAM)) and enzyme activities (ACC-oxidase (ACO) and ACC-synthase (ACS)) were assessed.

**Results:**

All tissues showed a similar climacteric pattern in ethylene productions, but with a different amplitude. Profound differences were found between tissue types at the metabolic and enzymatic level. The pericarp tissue produced the highest amount of ethylene, but showed only a low ACC content and limited ACS activity, while the locular gel accumulated a lot of ACC, MACC and SAM and showed only limited ACO and ACS activity. Central tissues (septa, columella and placenta) showed a strong accumulation of ACC and MACC. These differences indicate that the ethylene biosynthesis pathway is organized and regulated in a tissue specific way. The possible role of inter- and intra-tissue transport is discussed to explain these discrepancies. Furthermore, the antagonistic relation between ACO and E8, an ethylene biosynthesis inhibiting protein, was shown to be tissue specific and developmentally regulated. In addition, ethylene inhibition by E8 is not achieved by a direct interaction between ACO and E8, as previously suggested in literature.

**Conclusions:**

The Ethylene biosynthesis pathway and E8 show a tissue specific and developmental differentiation throughout tomato fruit development and ripening.

## Background

**Figure 1:**
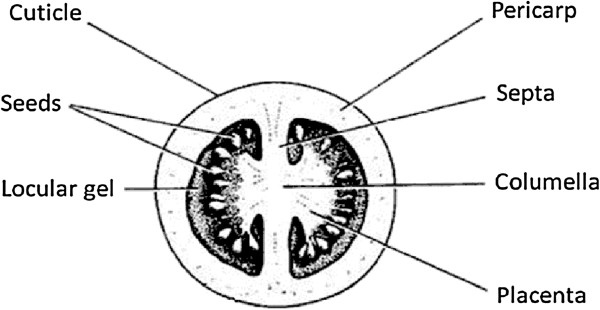
Schematic cross-section of a tomato fruit showing two locules and the different tissues.

Ethylene is the plant hormone that regulates amongst others climacteric fruit ripening. Over the years, tomato (*Solanum lycopersicum* L.) has become the model crop to study fleshy fruit ripening [1] and shows a far more complex tissue specialization compared to other well studied climacteric fruit like apple, avocado, persimmon or banana. A tomato fruit (Figure 1) is composed of several locules in which the seeds are located, protected by the surrounding locular gel. The seeds are attached to the placenta by the funiculus. The placenta tissues are interconnected by the firmer inner columella tissue. This columella tissue connects the fruit with the plant through the pedicel. Each locule is separated by two septa connecting the columella with the outer pericarp tissue, which is surrounded by the fruit cuticle.

Earlier work has well characterized the biochemical and molecular organization and regulations of the ethylene biosynthesis pathway. Ethylene is synthesized from its precursor 1-aminocyclopropane-1-carboxylic acid (ACC) by ACC oxidase (ACO) in the presence of oxygen [2,3]. ACC can also be converted into the biological inactive malonyl-ACC (MACC) by ACC-N-malonyltransferase [4,5] or into minor derivates like 1-γ-glutamyl-ACC (GACC) [6] or jasmonic acid-ACC (JA-ACC) [7]. ACC itself is made from S-adenosyl-L-methionine (SAM) by ACC synthase (ACS) [8].

In the past, tomato fruit biology has almost exclusively focused on pericarp tissue [9]. Little is known about the physiology and biochemistry of other tomato fruit tissues, let alone their interdependencies. Some emphasis to unravel tissue specialization in tomato fruit has already been done, focusing on e.g. DNA methylation [10], polyamine metabolism [11], malate and fumarate metabolism [12], sugar metabolism [13]–[16] and photosynthesis [17]. Besides these targeted studies, some large scale omics studies have mapped differences between tomato fruit tissues. Tissue specific screenings were done by transcriptomics and metabolomics of the primary and secondary metabolism [18]–[20]. Recently, [9] analyzed the transcriptome of the main pericarp cell types (outer and inner epidermal cells, collenchymas, parenchyma and vascular cells) leading to the discovery of an inner pericarp cuticle.

With respect to the ethylene metabolism, tissue specific analyses are largely lacking, although previous work has shown that locular gel breakdown precedes actual fruit ripening and pericarp softening [21,22]. The locular gel produces ethylene prior to other tissues [21] and it responds to external ethylene comparable with pericarp tissue [23]. At breaker stage, gel and columella tissue produce more ethylene than outer pericarp tissue leading to the conclusion that tomato fruit start to ripen from the inside out [21]. It was also demonstrated that MACC formation by ACC-N-malonyltransferase was most active in orange pericarp tissue and mature seeds [24]. GACC formation was shown to be most active in pericarp and placenta tissue of ripe tomato and in seeds of breaker fruit [6].

Our previous work displayed an extensive targeted systems biology investigation of the ethylene metabolism in pericarp tissue, revealing a novel regulatory mode during postharvest where ACO is the rate limiting step [25]. In the broader concept of a systems biology approach, we present a tissue specific investigation of the ethylene biosynthesis pathway in tomato. All major fruit tissues were profiled throughout fruit development, climacteric ripening and postharvest storage. Intermediate metabolites (SAM, ACC and MACC) were quantified along with the activity of ACS and ACO and the tissues specific ethylene production. This detailed screening allowed a comprehensive 3D interpretation of the ethylene metabolism, identifying many tissue specific biochemical differences within the fruit. Our data clearly showed that the ethylene metabolism is differentially organized and regulated in tomato.

## Results

### Characterization of fruit ripening physiology

**Figure 2:**
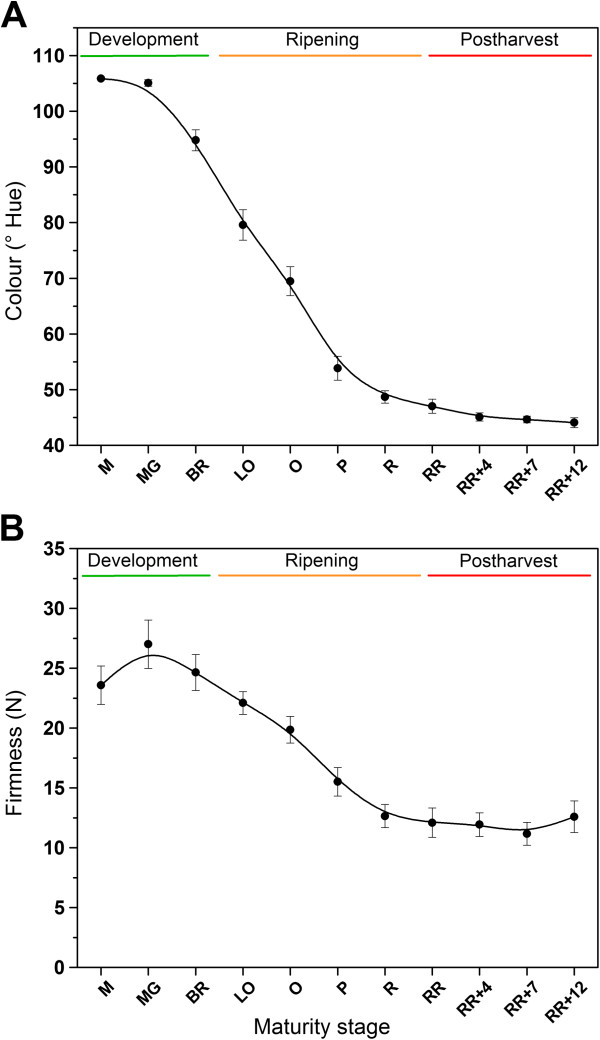
**Characterization of the different tomato fruit developmental stages (fruit development, ripening and postharvest storage). (A)** Fruit color (hue in °) and **(B)** firmness (N). Error bars represent the standard deviation of 12 biological replicates. The trend is visualized by a spline. Fruit maturity stage annotations: M. Medium sized fruit; MG. Mature Green fruit; BR. Breaker fruit; LO. Light Orange fruit; O. Orange fruit; P. Pink fruit; R. Red fruit; RR. Red Ripe fruit; RR + X. Red Ripe fruit + X days of postharvest storage.

**Figure 3:**
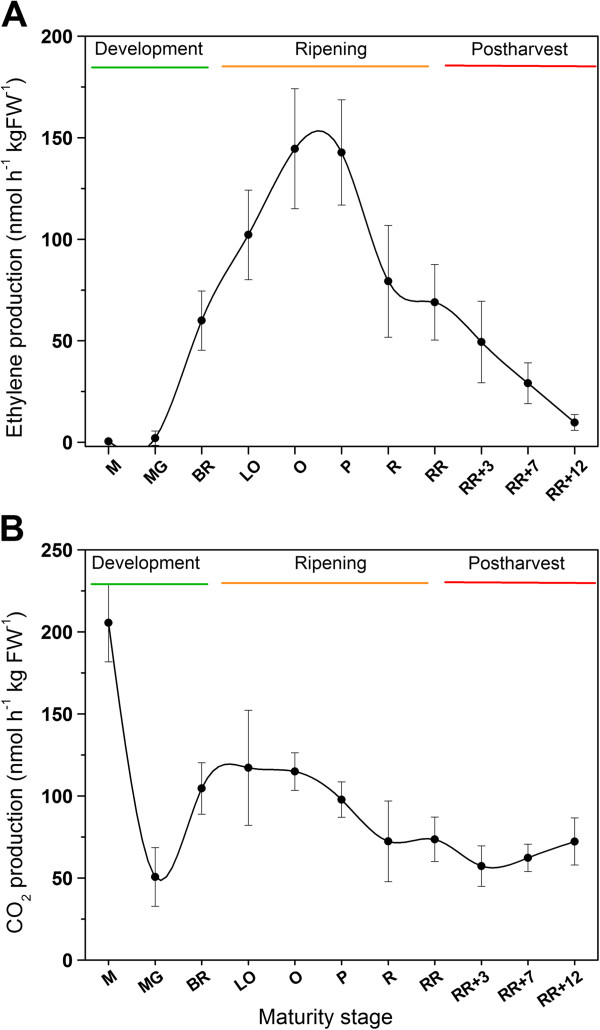
**Characterization of the climacteric behavior of tomato fruit. (A)** Ethylene production (nmol h^-1^ kg FW^-1^) and **(B)** respiration rate (nmol h^-1^ kg FW^-1^). Error bars represent the standard deviation of 12 biological replicates. The trend is visualized by a spline. M. Medium sized fruit; MG. Mature Green fruit; BR. Breaker fruit; LO. Light Orange fruit; O. Orange fruit; P. Pink fruit; R. Red fruit; RR. Red Ripe fruit; RR + X. Red Ripe fruit + X days of postharvest storage.

Fruit color, firmness, reparation and ethylene production of the intact fruit were measured in order to characterize the different tomato fruit maturity stages. Figure 2 and Figure 3 show the results for these traits during fruit development, climacteric ripening and postharvest storage. Fruit hue color ranged from green (approximately 107°) to red (approximately 45°). The strongest decline in hue corresponding to fruit ripening started from the breaker stage on, until the red ripe stage. During postharvest storage fruit color did not change anymore. Fruit firmness dropped from the breaker stage until the red ripe stage, correlating well to the ripening process. During postharvest storage firmness remained unaltered. Fruit respiration rate (CO_2_ production) was very high in small developing fruit, but rapidly declined. At the onset of ripening (breaker stage), respiration rate increased transiently, corresponding to the climacteric behavior of the fruit. Fruit ethylene production was low during fruit development, which corresponds to the basal ethylene production level of the ethylene auto-inhibitory system 1. From the breaker stage on, fruit ethylene production increased drastically which corresponds to the autocatalytic ethylene production level of system 2. During the post-climacteric stages ethylene production dropped again gradually.

### Characterization of wound ethylene

**Figure 4:**
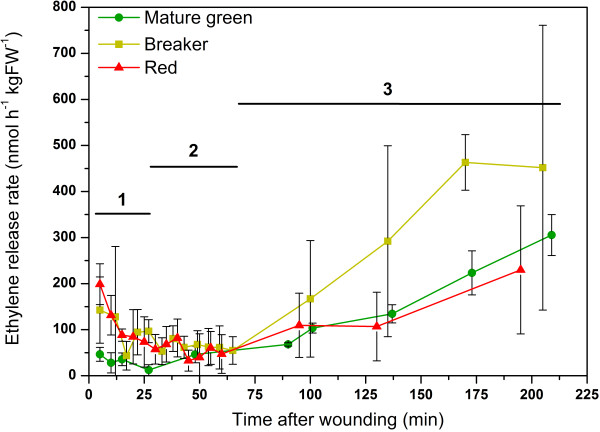
**Ethylene production after wounding.** Ethylene release rate (nmol h^-1^ kgFW^-1^) of sliced tomatoes, represented by a mixture of all tissue types, for mature green (green), breaker (yellow) and red (red) fruit for a period of 200 min after wounding. Three different phases are observed: (1) Ethylene diffusion phase; (2) Autonomous ethylene production phase; (3) Wound induced ethylene production phase. Error bars represent the standard deviations of five biological replicates.

In order to study the autonomous ethylene production level of the different tissues, fruit needed to be dissected which in turn triggers the wound ethylene response. To exclude the additional wound ethylene from the autonomous tissue specific ethylene production level, one needs to know when wound ethylene sets in and becomes observable. Figure 4 shows the ethylene release rate after cutting fruit of three different maturity stages (mature green, breaker and red). This graph can be divided into three different phases. The first phase (1) is characterized by a decline in ethylene release rate. This initial drop can be explained by a reduced diffusion gradient in the injured cells/tissues. The internal ethylene levels are quickly dropping because the main gas diffusion barrier was removed due to cutting of the fruit. Red and breaker fruit showed a stronger decline in ethylene release rate compared to mature green fruit, probably because these fruit initially contained more dissolved ethylene that consequently can diffuse out of the tissue after wounding. From 25 min to 65 min after wounding, the ethylene release rate was more or less constant. This second phase (2) corresponds to the autonomous ethylene production level of the sliced tomato fruit. This graph represents the overall ethylene production level of all tissues together, since whole fruit were cut in small pieces. At 65 min after wounding ethylene production slowly increased again. This third phase (3) is characterized by the wound-induced ethylene response. Note that breaker fruit had a higher wound ethylene production rate compared to mature green or red wounded tomatoes. Breaker fruit also showed more variation in their ethylene production rate, probably because this group is in transition from immature green to ripening fruit. This graph clearly shows that it lasts up to one hour before wound ethylene production starts. It also shows that measuring ethylene production levels immediately after wounding can be misleading. Therefore all subsequent experiments were done during the autonomous ethylene production phase: 25 – 65 min after wounding.

### Data normalization

**Figure 5:**
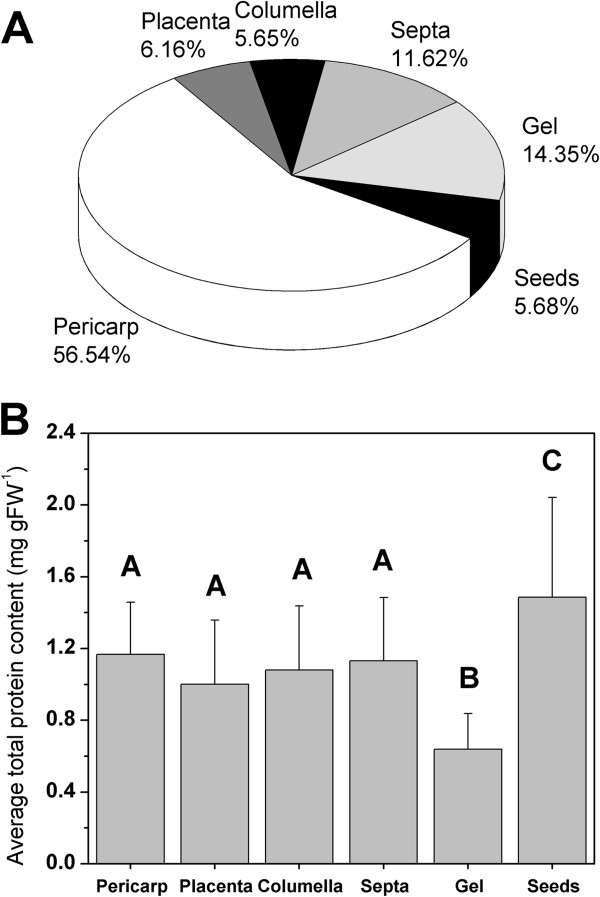
**Tissue distribution percentage and protein content. (A)** Average percentage fresh weight of the various tissue types of a tomato fruit and **(B)** the tissue specific protein content in mg protein gFW^-1^. Values represent the average over all maturity stages and error bars represent standard deviation. Statistical significant differences (P < 0.05) between treatments are indicated by different letters.

Since different tissues contain unequal amounts of water and dry matter, one commonly normalizes biochemical data by expressing the measured values relative to the total protein content of the tissue. Figure 5 shows the average percentage contribution of the various tissues to the fresh weight of a tomato fruit and the average protein content of the different tissues (averaged over all maturity stages). It is clear that pericarp is the most abundant tissue in a tomato fruit, with seeds and columella being the least abundant. All tissues have more or less the same protein content (ranging between approximately 1.0-1.5 mg protein gFW^-1^) except for the gel, which contains around half the amount (aproximately 0.7 mg protein gFW^-1^). This tissue specific protein content is used to normalize the collected metabolic and enzymatic data.

### Ethylene production is tissue specific

**Figure 6:**
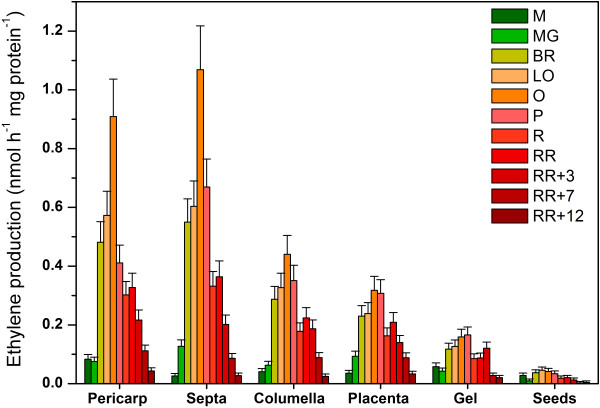
**Ethylene production of the different tissues.** Ethylene production (nmol h^-1^ mg protein^-1^) for the different tomato fruit tissues during fruit development, climacteric ripening and postharvest storage. Error bars represent the standard deviation of 3 biological replicates. M. Medium sized fruit; MG. Mature Green fruit; BR. Breaker fruit; LO. Light Orange fruit; O. Orange fruit; P. Pink fruit; R. Red fruit; RR. Red Ripe fruit; RR + X. Red Ripe fruit + X days of postharvest storage.

Ethylene production of the different tissues was measured during the autonomous ethylene production phase. Since not all tissues have equal dry matter content, ethylene production rates were expressed in relation to the tissue’s protein content instead of their fresh weight. Figure 6 shows the ethylene production (in nmol/h mg protein) of each tomato fruit tissue examined. Although normalized the same way, the individual tissues produced substantially less ethylene than the entire fruit (see Figure 3). All tissues showed a climacteric ethylene production pattern, being low during fruit development, rising autocatalytically during ripening and declining during post-climacteric ripening and postharvest storage. The pericarp and the septa showed the highest climacteric rise in ethylene production rate, while the placenta and the columella showed an intermediate increase. The gel showed the lowest climacteric rise while the seeds remained more or less at their basal ethylene production level. During the final postharvest stages the ethylene production rate of all tissues declined to similarly low levels.

### Characterization of ethylene biosynthesis metabolites (SAM, ACC and MACC)

**Figure 7:**
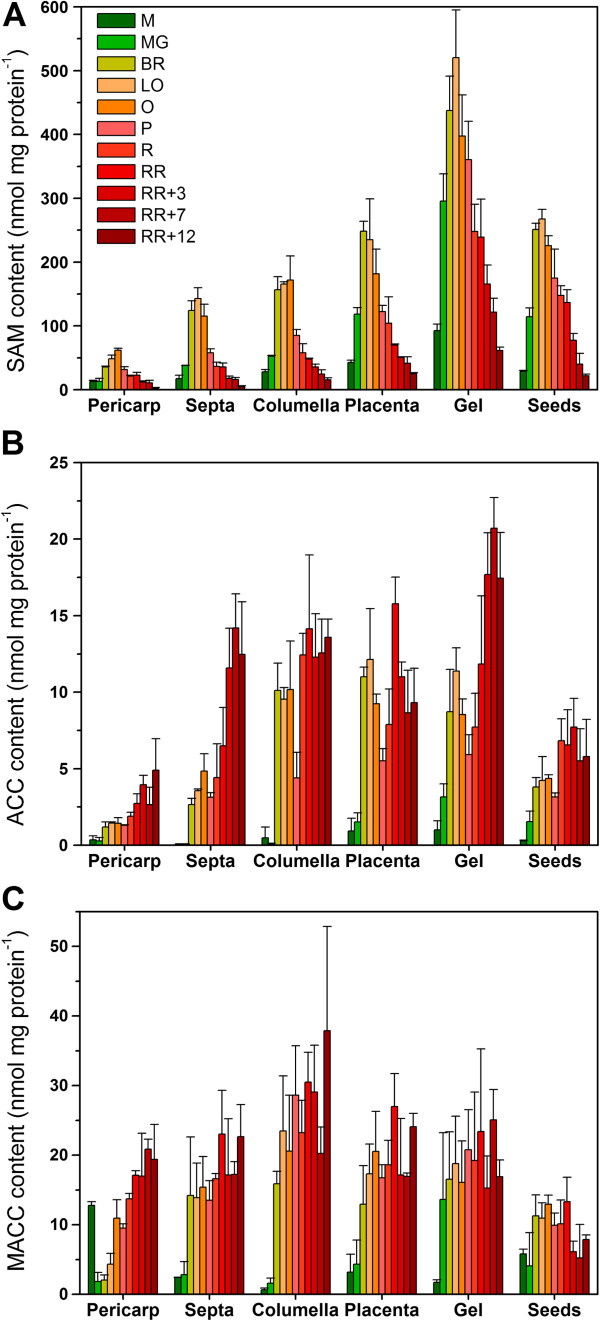
**Metabolite content of the different tissues. (A)** SAM content (nmol mg protein^-1^), **(B)** ACC content (nmol mg protein^-1^) and **(C)** MACC content (nmol mg protein^-1^) for the different tomato fruit tissues during fruit development, climacteric ripening and postharvest storage. Error bars represent the standard deviation of 3 biological replicates. M. Medium sized fruit; MG. Mature Green fruit; BR. Breaker fruit; LO. Light Orange fruit; O. Orange fruit; P. Pink fruit; R. Red fruit; RR. Red Ripe fruit; RR + X. Red Ripe fruit + X days of postharvest storage.

Besides ethylene production, all intermediate metabolites of the pathway were quantified during fruit development, ripening and postharvest storage (Figure 7). All tissues showed a similar metabolic profile except for SAM. SAM content increased just prior to ripening and dropped again at the pink-red stage. Changes in SAM content always preceded changes in ethylene production. SAM levels were highest in the gel, being around 10 times higher than SAM levels in the pericarp. Seeds, septa, columella and placenta also contained substantially higher amounts of SAM compared to the pericarp.

ACC and MACC levels were very low during fruit development, and started to increase at the onset of ripening. Both metabolites continued to increase in all tissues reaching their highest levels during postharvest storage. ACC was most predominant in the locular gel (like SAM) and the lowest in the pericarp tissue. MACC levels were much higher (around 4 times for e.g. pericarp tissue) than ACC levels. MACC was most predominantly present in the gel and the columella, but the pericarp, septa, placenta and gel also contained high amounts of MACC. The seeds showed the lowest levels of MACC.

### Characterization of enzyme activity (ACO and ACS)

**Figure 8:**
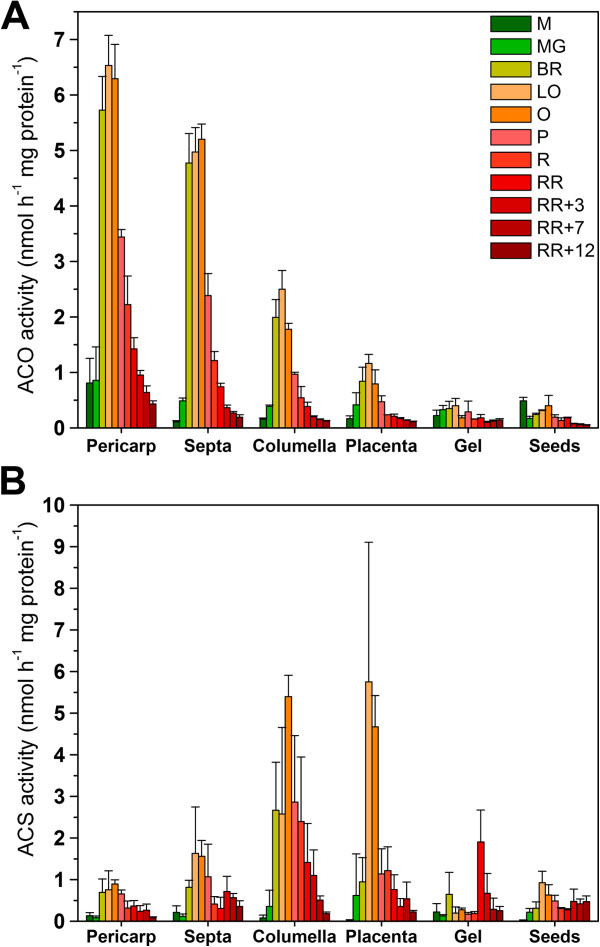
**Ethylene biosynthesis enzyme activity of the different tissues. (A) ***In vitro* ACO activity (nmol h^-1^ mg protein^-1^) and **(B) ***in vitro* ACS activity (nmol h^-1^ mg protein^-1^) for the different tomato fruit tissues during fruit development, climacteric ripening and postharvest storage. Error bars represent the standard deviation of 3 biological replicates. M. Medium sized fruit; MG. Mature Green fruit; BR. Breaker fruit; LO. Light Orange fruit; O. Orange fruit; P. Pink fruit; R. Red fruit; RR. Red Ripe fruit; RR + X. Red Ripe fruit + X days of postharvest storage.

To obtain more information on how metabolites are synthesized and consumed, *in vitro* enzyme activity was measured for both ACO and ACS in all different tissues during fruit development, ripening and postharvest storage (Figure 8). ACO activity showed a climacteric pattern comparable to the *in vivo* ethylene production (see Figure 6), in other words, a low activity during fruit development, a strong increase at the onset of ripening and a gradual decrease in activity during further ripening and postharvest storage. Pericarp tissue showed the highest ACO activity followed by the septa and the columella. The gel and the seeds hardly showed any ACO activity, although the gel did show some *in vivo* ethylene production.

ACS activity started to increase from the breaker stage on and was maximal around the light orange – orange stage. The pericarp, the seeds and the gel showed only a low ACS activity during ripening, while the septa showed an intermediate ACS activity. The inner tissues like the placenta and columella showed the highest ACS activity, which was around six times higher than the pericarp tissue.

### Western blotting reveals an antagonistic relation between ACO and E8

**Figure 9:**
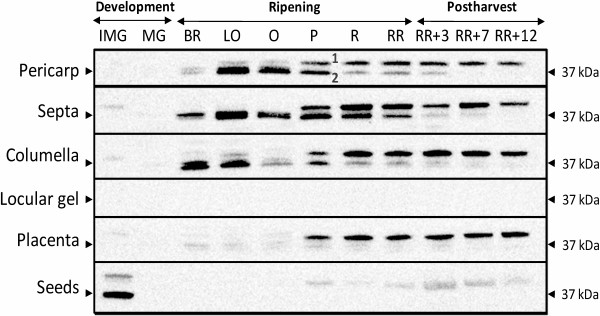
**Western blots of the different tomato fruit tissues.** ACO Western blots of the different tissues during fruit development, climacteric ripening and postharvest storage, developed with the custom made anti-ACO antibody. Two bands are observed: E8 (1) and ACO (2). The 37 kDa marker is indicated by an arrowhead.

Because ACO was found to be the rate limiting step during post-climacteric ethylene production [25], we decided to further study the tissue specific ethylene biosynthesis at the protein level by doing Western blots against ACO (Figure 9). The antibodies used in this assay were designed against a conserved peptide, present in four ACO isoforms (ACO1-4). Remarkably, two clear bands were observed (indicated with number 1 and 2 on the blot). The lower band (2) matches the predicted protein mass of ACO, while the upper band (1) is located around 10 kDa higher. These two discrete bands were also observed when Western blots were developed with commercial anti-ACO antibodies and also for tomato leaf and apple fruit tissue (Additional file 1: Figure S1). In order to identify the two bands, peptide sequencing by MALDI-TOF/TOF mass spectrometry was performed on different zones around the 37 kDa region of a SDS-PAGE (Additional file 1: Figure S2). This analysis led to the identification of ACO as being the lower band (2), and the previously described E8 protein as being the upper band (1).

With this knowledge, the Western blots presented in Figure 9 are further analyzed. ACO abundance is correlated with ACO *in vitro* activity in all tissues and throughout the entire developmental period. At some stages it is even possible to see two bands right on top of each other (e.g. columella at breaker stage), which most likely represent two different ACO isoforms.

Western blot analysis also allowed observing that E8 shows an antagonistic relation with ACO throughout fruit development and ripening. Whenever ACO abundance was declining, E8 abundance was increasing (during the postharvest stages), with a slight overlap around the pink stage. Interesting to observe was that E8 is highly abundant in the placenta, while ACO abundance is hardly observed and ACO activity is minimal. The seeds that did not produce any significant amounts of ethylene showed only a little abundance of E8. The gel on the other hand did not show any observable amount of ACO nor E8.

### E8 shows no direct inhibitory effect on ACO activity

In order to further investigate the antagonistic relation between E8 and ACO abundance/activity and in particular ethylene production, an overexpression study was performed. Both for *ACO1* and *E8* the full length cDNA sequences extended with a C-terminal His-tag, were overexpressed in *E. coli* (BL21). After IPTG induction, both proteins were purified from total cell lysates using Ni-NTA columns and their purity and identity was checked on a coomassie stained SDS-PAGE (Additional file 1: Figure S3). The purified proteins were also double checked by MALDI-TOF/TOF for further identification and Western blot for antibody specificity (Additional file 1: Figure S4). All these results indicate that both ACO1 and E8 are indeed overexpressed and highly purified. The antibodies used in this study interact with both ACO and E8 (Additional file 1: Figure S5), although both proteins show only limited amino acid sequence identity with each other (34%; Additional file 1: Figure S6).

**Figure 10:**
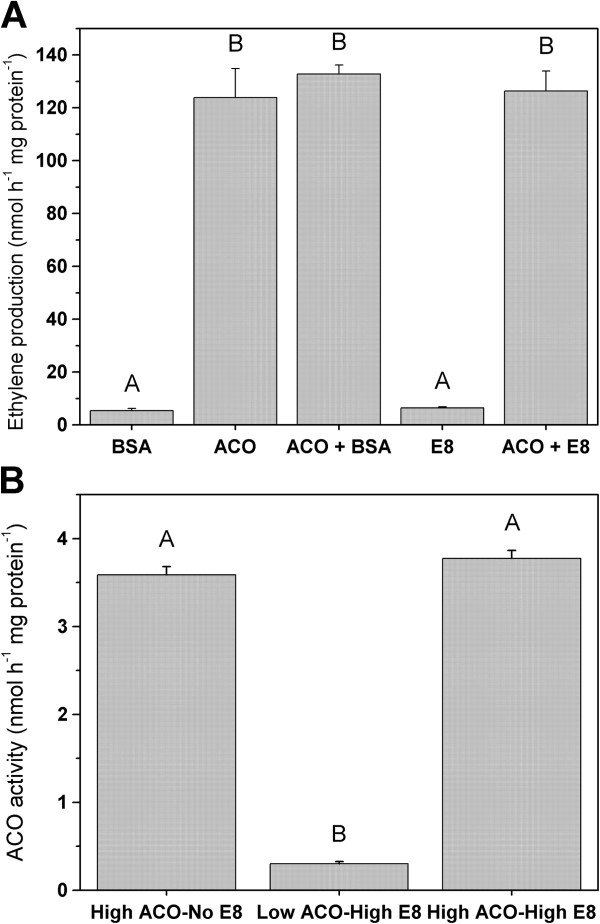
**Effect of E8 on ACO activity and ethylene production. (A)** Ectopically overexpressed *in vitro* ACO activity was not altered by the addition of ectopically overexpressed E8. **(B)** The effect of a possible ACO activity inhibition was also evaluated in pericarp enzyme extracts, by mixing a sample with no E8 abundance and high ACO activity (left) with a sample containing a high E8 abundance and low ACO activity (middle), resulting in no inhibition of ACO activity (right). Error bars represent the standard deviation of 3 replicates. Statistical significant differences (P < 0.05) between treatments are indicated with A and B.

An *in vitro* assay showed that E8 has no inhibiting effect on ethylene production by ACO (Figure 10). This is the case for both the purified ectopically expressed enzyme as for an extracted protein sample of tomato pericarp. The Western blot data combined with these activity assays, indicate that E8 apparently shows an antagonistic relation with ACO, but it is unlikely that E8 influence ethylene production through ACO-mediated protein interactions. The exact biochemical function of E8 remains to be elucidated, and is further discussed below.

## Discussion

### Tissue specific heat-plot visualization of the ethylene metabolism

**Figure 11:**
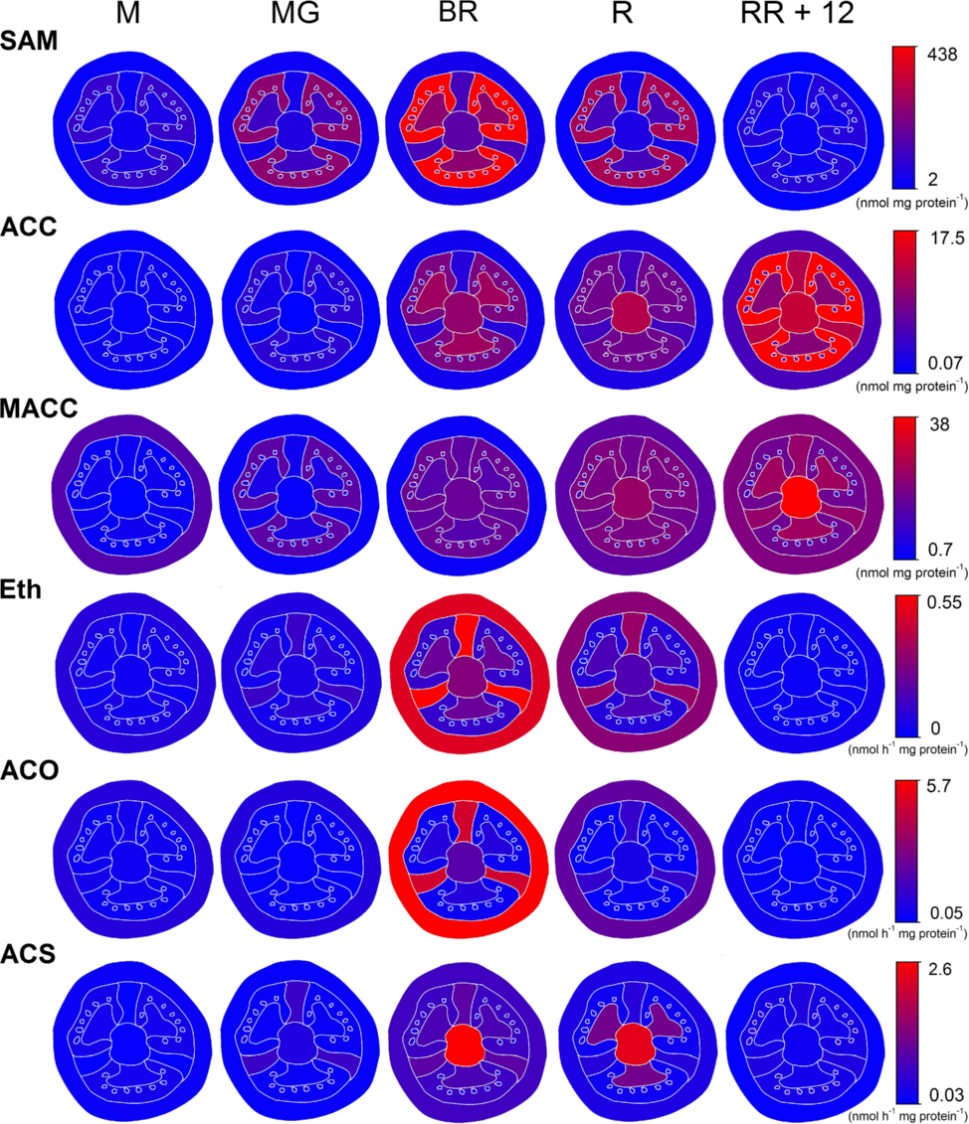
**Heat plot representation of a cross-section of a schematic tomato fruit.** Visualization of the evolution of the ethylene metabolism for the different tissues for SAM, ACC and MACC content, *in vivo* ethylene production (Eth), *in vitro* ACO activity and *in vitro* ACS activity. Individual colors represent the amount of metabolite or enzyme activity for all maturity stages. Fruit maturity stage annotations: M. Medium sized fruit; MG. Mature Green fruit; BR. Breaker fruit; R. Red fruit; RR + 12. Red Ripe fruit + 12 days of postharvest storage.

In order to summarize the major changes of the fruit ethylene metabolism, a heat-plot like visualization was made for the different tissues for five major developmental stages (small, mature green, breaker, red, RR + 12). This visualization (Figure 11) allows a direct interpretation of each metabolite or enzyme activity for each individual tissue with respect to the neighboring tissues. Ethylene production and ACO *in vitro* activity are closely correlated with each other. This means that ethylene is predominantly produced in the pericarp tissue, although its precursor metabolites ACC and SAM show only a low content in the pericarp. SAM is mainly located in the gel and is highly abundant during the mature green stage, just prior to the initiation in ethylene production. ACC content is also highly present in the gel. MACC is mainly located in the gel and the other internal tissues (columella, placenta and septa) and only accumulates in the pericarp towards the end of the postharvest storage period. Ethylene production seems to be less associated with ACS activity which mainly takes place in the central tissues (columella and septa) during ripening and in the seeds during the final postharvest storage stages. Overall, Figure 11 illustrates the strong tissue specific organization of the ethylene metabolism in tomato fruit.

### Ethylene metabolism is organized in a tissue specific manner

By selectively profiling all ethylene biosynthesis intermediates and enzyme activities, the internal ethylene metabolism of ripening tomato fruit was fully characterized. In order to synthesize ethylene, a cell requires substrate (ACC and SAM), the necessary enzymes (ACO and ACS) and other essentials like co-factors (Fe^2+^ and pyridoxal-5-phosphate), activators (bicarbonate) and co-substrates (ascorbic acid and oxygen). It is clear from the data that pericarp tissue produces the most ethylene (both *in vivo* and *in vitro*). Although pericarp tissue has a high ACO activity, it only has a limited ACS activity and the lowest levels of precursors (ACC and SAM). This points to the fact that all ACC formed by ACS in the pericarp is quickly turned into ethylene, confirming ACS as the rate limiting step of ethylene biosynthesis as stated numerous times before (e.g. [26]). It is rather particular that the pericarp tissue produces the highest amount of ethylene, while it has the lowest amount of ACC and ACS-activity. It is possible that pericarp tissue just accumulates less ACC, because it has a high ACO activity, while the other tissues can accumulate more ACC due to their higher ACS activity (e.g. placenta and columella), as they produce less ethylene, yet this does not explain the low ACS activity observed in pericarp tissue. Perhaps ACC is supplied from another tissue (e.g. gel) to the pericarp in order to achieve such high rates of ethylene synthesis. The pericarp also shows a low MACC content compared to the other tissues, which indicates that the major part of ACC is used for ethylene biosynthesis and not for MACC formation. These observations suggest that the level of ACC is kept just high enough in the pericarp to ensure sufficient ethylene production. All in all, these discrepancies demonstrate that the ethylene metabolism is differentially regulated in different tissue types.

The locular gel, on the other hand, hardly showed any ACO and ACS activity, although it contains high amounts of intermediates (ACC and SAM). This indicates that most likely metabolites originate from a different tissue and are accumulating in the gel. Perhaps the gel functions as some kind of storage tissue, receiving excess metabolites from certain surrounding tissues (like e.g. the placenta), and supplying metabolites to other demanding tissues (like e.g. the pericarp).

The septa, the columella and the placenta all contain intermediate amounts of SAM and ACC and they show a rather high ACS activity. Thus the eventual rate of ethylene biosynthesis seems to be determined by the amount of ACO. Indeed, an intermediate ACO activity in the septa and the columella results in an intermediate *in vivo* ethylene production, while the lower ACO activity in the placenta is reflected in a lower *in vivo* ethylene production, in contrary to the thigh ACS activity in the placenta. These data suggest that ACO might be the controlling and/or rate limiting step in these tissues.

It is clear from the results that the ethylene metabolism is organized tissue specifically, as such that each tissue type has a distinct metabolic/enzymatic profile related to ethylene biosynthesis. This differential regulation most likely matches the specific physiological function of each individual tissue. Nonetheless, all tissues show a similar climacteric pattern in ethylene production throughout fruit development, yet with a different amplitude. This illustrates that, although there are tissue specific differences in the ethylene metabolism, the developmental cues of fruit ripening are programmed in each tissue.

### Antagonistic relation between ACO and E8 is conserved throughout different tissues and fruit development

The antibodies in our study showed cross-reactivity with the E8 enzyme, uncovering an antagonistic relation with ACO abundance. E8 was previously identified as an ethylene inducible gene in tomato [27]. Its expression was induced by ripening and enhanced by an ethylene treatment in a dose–response manner [28]. Studies with E8 antisense lines showed an absence of E8 protein during ripening, which resulted in an increase in ethylene production [29,30]. These results led to the conclusion that E8 is ethylene and ripening induced and is a negative regulator of ethylene biosynthesis and/or tomato fruit ripening.

Our results have demonstrated that there is a developmental and antagonistic relation between ACO abundance and E8 abundance. Whenever ACO abundance is declining during ripening, E8 abundance is increasing. This increase in E8 abundance also coincides with the decline in ethylene production, confirming the negative relation between E8 and ethylene production, as previously stated in literature. Furthermore our results have shown that certain tissues which show only limited amount of ethylene production (e.g. seeds, placenta and columella), all show a high content of E8, suggesting that E8 also negatively influences ethylene production in a tissue specific way.

These results combined with the fact that both proteins are 2-oxoglutarate-dependent dioxygenases [31] and that both enzymes contain leucine zippers, might suggest a direct protein interaction between ACO and E8. Nonetheless, both enzymes only show 34% amino acid sequence similarity (Additional file 1: Figure S6). In an attempt to further characterize this antagonistic relation, both ACO and E8 were overexpressed and purified. *In vitro* enzymatic assays revealed that there was no inhibition of ethylene synthesis by ACO in the presence of E8, and that E8 does not produce any ethylene from itself in the conditions tested. This study indicates that most probably ACO and E8 show no direct interaction, in contradiction to previous suggestions in literature [30]. Perhaps the negative effect of E8 on ethylene production is realized by another indirect regulation or through a metabolic feedback. E8 is a member of the dioxygenase enzyme family, and like many dioxygenases E8 might be involved in the biosynthesis route of a secondary metabolite. Perhaps such a secondary metabolite originating from an E8 mediated anabolism, could have a profound effect on ethylene biosynthesis. Although the exact biochemical function of E8 remains to be elucidated, our results suggest that there is no direct interaction between ACO and E8 and that the antagonistic relation between E8 and ethylene production is tissue and developmentally regulated in tomato.

### Inter-, intra-, and extracellular translocation or phloem and xylem mediated transport of ACC might regulate local ethylene biosynthesis

A measured metabolic concentrations and/or enzyme activity is a steady state observation which is the net sum of synthesis, consumption and transport. This last term of transport is often neglected. Metabolite transport might clarify some discrepancies observed in this study between the measured metabolites and their corresponding enzymes. For example, the locular gel contains high amount of metabolites (SAM, ACC and MACC) but only shows very little ACO and ACS activity. Perhaps metabolites from other tissues migrate towards the gel where they are stored (or redirected to other tissues). The pericarp tissue on the other hand showed only a limited ACS activity, while producing the highest amount of ethylene. Perhaps ACC is supplied to the pericarp originating from other tissues like for example the gel? Both hypotheses oblige the cell to posses the capability of ACC transport (active or passive).

Local transport of metabolites (and/or proteins) can be intracellular (mainly passive diffusion either or not facilitated by cytoplasmatic streaming) or intercellular (via symplastic transport through plasmodesmata or via apoplastic transport) [32]–[34]. Long-distance transport is achieved through the phloem (of both metabolites and macromolecules) and the xylem (mainly of water, sugars, ions, amino acids and hormones) [35,36]. Long distance transport of ACC from the roots to the aerial parts is a well-characterized response of tomato plants suffering from root stress (salinity, water deficit and hypoxia) [37]–[39]. This acropetal transport requires specific xylem loading and unloading of the highly polar non-protein amino acid ACC. Phloem mediated ACC transport was also observed in cotton plants [40]. Intracellular passive and active ACC transport across the tonoplast was also observed [41,42]. The exact ACC loading mechanism and the structural characterization of these ACC transporters remain to be discovered. All together, these observations suggest that the cell possesses multiple tools to accommodate ACC transport from one tissue to the other. These potential transport systems would provide the fruit with an additional regulatory mechanism to control ethylene production levels in certain parts of the fruit during certain developmental stages.

### Can SAM and MACC transport also regulate ethylene biosynthesis?

A similar reflection can be made for the malonyl derivate of ACC. The importance of this metabolite is conserved throughout the entire fruit, as our results have shown that MACC is very abundant in all tissues analyzed. These results also confirm the general belief that MACC is an end product and can thus easily accumulate [26]. Note, that the assay used in this study did not discriminate between MACC and other derivates like GACC and JA-ACC. These last derivates are poorly characterized and comprise only a small moiety of the pool of ACC derivates. Nonetheless, the importance of these derivates might be underestimated. Additionally, the reverse reaction of MACC formation (MACC hydrolysis) was observed twice in plants [43,44], providing a potential mechanism to control ethylene biosynthesis. The fact that MACC might be an end product was also supported by the observation that MACC could be translocated from the cytosol into the vacuole and back by ATP-mediated tonoplast carriers [41,45,46]. Perhaps these or similar processes can control the amount of MACC transported in between different tissues.

Less is known about SAM. Although this important molecule serves multiple pathways, it is often neglected in many ethylene related studies. Besides the biosynthesis of ethylene, SAM mainly participates in the biosynthesis of polyamines and numerous transmethylation reactions [47]. This manifold usage requires a stringent regulation of the SAM pool through synthesis, consumption, recycling and perhaps translocation [48]. SAM specific transport proteins were identified in Arabidopsis to ensure SAM translocation from the cytosol to the mitochondria and the chloroplasts [49]. Whether this subcellular delocalization of SAM in turn can have an effect on ethylene biosynthesis, or if SAM can also be transported between different tissues, remains to be investigated.

## Conclusions

In an attempt to better understand ethylene biosynthesis in ripening tomato, the ethylene biosynthesis pathway was analyzed for different fruit tissues: pericarp, septa, locular gel, placenta, columella and seeds. The results have demonstrated that all tissues show a similar climacteric pattern in ethylene production, but large differences were observed for intermediate metabolites and enzymes. Locular gel produced only limited amount of ethylene but accumulated a high content of intermediates (ACC, MACC and SAM). Central tissues (septa, placenta and columella) mainly accumulated ACC and MACC. Pericarp tissue showed the highest ethylene production during ripening, but contained only a limited amount of intermediates and surprisingly showed only a minor ACS activity. Furthermore the antagonistic relation between ACO and E8 was characterized. It was also shown that both proteins do not interact in order to inhibit ethylene production. Finally, inter- and intra-tissue transport is discussed to accommodate the tissue specific discrepancies observed, which may act as a potential mechanism to control fruit ethylene production.

## Methods

### Plant material

Tomato fruit (*Solanum lycopersicum* L. ‘Bonaparte’) of different maturity stages were harvested from the Research Station of Vegetable Production of both Sint-Katelijne-Waver and Hoogstraten (Belgium during the months March-May 2013. Plants were cultivated hydroponically on rockwool under natural lightning and were kept at optimal temperature (23/21°C day/night) and humidity (70% RH) to obtain commercial yield. Twelve fruit of each maturity stage (medium size, M; mature green, MG; breaker, BR; light orange, LO; orange, O; pink, P; red, R and red ripe, RR) were harvested for immediate analyses of fruit color, firmness, ethylene production and respiration rate (CO_2_ production) as described by [22,50]. Additionally, red ripe fruit were harvested for analysis after respectively 4, 7 and 12 days (12 fruit per stage) of postharvest storage at shelf life conditions (18°C and 80% RH).

The fruit from these batches were subsequently dissected, crushed in liquid nitrogen and stored at −80°C for further metabolic and enzyme activity measurements.

### Characterization of wound ethylene

A tissue specific characterization is only possible by dissecting the fruit. This destructive operation induces the wound ethylene response and should be taken into account in order to exclude the wound induced ethylene production from the autonomous tissue specific ethylene production capacity. A separate batch of five fruit for three different maturity stages (mature green, breaker and red) was harvested to asses this wound ethylene response. After harvest, each fruit was individually cut in small pieces so all different tissue types were mixed, leading to five biological replicates. From this tissue mixture, originating from one fruit and representing all tissues, 3 g fresh weight was incubated for 5 min in an airtight glass jar (20 mL) containing a septum. Ethylene in the headspace was assessed by gas chromatography (Compact GC, Interscience, Louvain-la-Neuve, Belgium) as described by [50]. After the ethylene measurement, the sample was briefly flushed with normal air and sealed again for 5 min. Ethylene levels in the headspace were continuously monitored at regular time intervals for a total period of 200 min after wounding with systematic flushing in between. This experiment allowed to characterize the timeslot during which the wound induced ethylene production has not yet commenced.

### Assessment of tissue specific ethylene production

To measure the tissue specific ethylene production, another batch of 12 fruit for each maturity stage was dissected and the different tissues were pooled per tissue type for each maturity stage. This pooling was done to have sufficient amount of material of each tissue to asses the ethylene production. This process was repeated 3 times in order to have 3 biological replicates. The tissue specific ethylene production was assessed in the wound ethylene free timeslot (see above). Ethylene production was measured for 3 g fresh weight of each tissue type. The tissue was incubated for 5 min in a 20 mL airtight glass jar containing a septum. Ethylene content in the headspace was measured as described by [50].

### Metabolite and enzyme activity measurements

The original batches of 12 tomatoes of each maturity stage that were first assessed for their entire fruit ethylene production, were subsequently dissected and the different tissues were flash frozen in liquid nitrogen and stored at −80°C. The tissues originating from 12 fruit were pooled in order to have sufficient material for all the biochemical analyses, and this was repeated 3 times in order to have 3 biological replicates. For each maturity stage and each tissue type, all metabolites (SAM, ACC and MACC) and enzyme activities (ACO and ACS) from the ethylene biosynthesis pathway were quantified. SAM was extracted and quantified by capillary electrophoresis (P/ACE-MDQ, Beckmann Coulter, Fullerton, CA, USA) in a glycine : phosphate buffer (300 : 50 mM, pH 2.5) as described by [51]. ACC and MACC content was measured exactly as described by [50].

The *in vitro* enzyme activity of ACO and ACS was also measured as described by [50] but for the ACO assessment the MOPS buffer was replaced by a 100 mM Tris buffer (pH 8.0), and the incubation time of the ACO assay was optimized to 15 min. Total protein content of the ACO and ACS extract was determined following the Bradford assay [52].

### Western blotting of ACO

Polyclonal antibodies were developed (GenScript, GE Healthcare, Piscataway, NJ, USA) against a consensus epitope for four ACO isoforms (ACO1 [UniProt P05116], ACO2 [UniProt P07920], ACO3 [UniProt P10967] and ACO4 [UniProt P24157] - CQDDKVSGLQLLKDE). For SDS-PAGE, 15 μg total protein content was loaded on a 12 wells 8–16% TGX Criterion precast gel (Bio-Rad, Hercules, CA, USA) and ran for 45 min at 180 V in Laemmli buffer. Subsequent electroblotting was carried out for 1 h 20 min at 100 V on a PVDF membrane (GE Healthcare) in the presence of transfer buffer (25 mM Tris, 140 mM glycine, 20% (v/v) methanol). The membrane was blocked for 1 h in TBS-T (25 mM Tris, 125 mM NaCl and 0.1% (v/v) Tween-20) containing 5% milk powder. After blocking, the membrane was incubated overnight at 4°C with primary antibody solution (1/1000 anti-ACO AB in TBS-T with 5% milk powder). Subsequently the membrane was washed 5 times for 5 min in TBS-T and secondary antibody (1/2000 Anti-Rabbit-HRP-linked AB; Cell Signaling Technologies Inc., Danvers, MA, USA) was incubated for 2 h at 4°C. Again the membrane was washed and subsequently enhanced chemoluminescence was performed with Clarity ECL western substrate (Bio-Rad) and detected with the ImageQuant LAS4000 system (GE Healthcare).

### Mass spectrometry identification of ACO and E8

On western blot two bands were visible around 37 kDa. To identify these bands MALDI mass spectrometry analyses were done on several zones around 37 kDa that were dissected from a coomassie stained gel. The cut out zones were subjected to *in gel* digestion using trypsin and extracted as described previously [53]. MALDI mass spectrometry analysis was performed on a 4800 MALDI TOF/TOF mass spectrometer (4800 Proteomics Analyzer, Applied Biosystems, Foster City, CA, USA). Measurements were executed in positive ion mode and the mass range was set between 900–3500 *m/z*. For each band, the 15 most intense ions were selected for MS/MS analysis. An exclusion list of peaks resulting from autodigestion of trypsin was used. The resulting peak lists were submitted to a Mascot Database Server (Version 2.2) for identification, supplemented with a tomato protein sequence database from NCBI. Additional masses of interest were subjected to MS/MS analysis for identification.

### Cloning, overexpression and purification of ACO1 and E8

ACO and E8 proteins were further investigated by overexpression. The full length cDNA of both genes (*ACO1* [NCBI ×04792] for ACO and *E8* [NCBI X13437]) were cloned into a pET28a vector (using XbaI and SalI) resulting in a fusion to a C-terminal His-tag. The plasmids sequences were verified by sequencing, and transformed into a BL21 (DE3) *E. coli* strain for protein overexpression. In total 500 mL cultures were grown at 35°C until an OD of 0.5-0.6 was reached. Then protein expression was induced by adding 1 mM IPTG and the cultures were further incubated for 3 h at 30°C. Cells were harvested by centrifugation for 15 min at 4800 × g at 4°C, and the pellet was washed in 15 mL of 50 mM Tris pH 8.0. The suspension was centrifuged again for 15 min at 4800 × g at 4°C. The pellet was subjected to lysis by dissolving the pellet in lysis buffer (4 mL per g cells) supplemented with 1 mg mL-1 lysosyme, 5 μg mL-1 DNase I and 10 μg mL-1 RNase. The suspension was subsequently sonicated on ice for 30 sec at 20% followed by 30 sec rest for a total period of 4 min. This was repeated three times. Then, the lystae was centrifugated at 10.000 × g for 40 min at 4°C, and the supernatants was stored at – 80°C for further purification.

The lysate was purified using Nikkel-NTA chromatographic columns on a UPLC system (AktaPurifier, GE Healthcare). The overexpressed proteins (both ACO and E8) were eluted with 80 mM imidazole in 20 mM phosphate and 0.5 M NaCl at pH 7.4. To verify the purity of the elution, the samples were run on a SDS-PAGE with coomassie staining. Additional peptide sequencing was done by MALDI TOF/TOF mass spectrometry (described above) to verify protein identification.

### Generation of heat-plots

In order to visualize the results in a tissue specific way, heat-plots of the main developmental stages were constructed. This allows a direct observation of the main metabolic and enzymatic differences in a developmental and tissue specific way. A text-image of a transversal section of a tomato fruit was generated with Microsoft Office^®^ Excel and recoloured with Image J [54]. Each tissue was given a value of a fixed color scale (0–255) corresponding to the measured value ranging between the minimum (0) and maximum (255) value of each dataset.

### Statistical analysis

Statistical differences were analyzed with the one-way ANOVA procedure using the Statistical Analysis Software (SAS Enterprise Guide 4.2; SAS Institute Inc.). Confidence intervals were set at 95%.

## Competing interests

The authors declare no competing interests.

## Authors’ contributions

Designed the study (B.V.d.P., M.L.A.T.M., M.P.D.P., B.M.N., A.H.G.). Performed biochemical analysis (B.V.d.P., N.V., C.S., I.B., I.M.). Performed mass spectrometry analysis (S.V., R.D., E.W.). Performed protein overexpression and purification (B.V.d.P., T.N., S.S., J.V.). Analyzed the data (B.V.d.P., M.L.A.T.M., M.P.D.P., B.M.N., A.H.G.). Drafted the manuscript (B.V.d.P., M.L.A.T.M., B.M.N., A.H.G.). All authors read and approved the final manuscript.

## Supplementary Material

Additional file 1: Figure S1 Additional Western blots to characterize the two bands. **Figure S2.** MALDI-TOF/TOF peptide analysis the two bands. **Figure S3.** Coomassie stained SDS-PAGE of the purified His-tagged ACO and E8 proteins. **Figure S4.** Identification of the purified ACO and E8 after overexpression. **Figure S5.** Sequence properties of the custom polyclonal anti-ACO antibody. **Figure S6.** Sequence alignment between tomato ACO1 and E8. Click here for file

## Aknowledgements

We thank G. Pittoors from Pittoma N.V. (Belgium) and the Research Station of Vegetable Production of both Sint-Katelijne-Waver and Hoogstraten for providing plant material. We also acknowledge the Flanders Centre of Postharvest Technology (VCBT) for collaborating and providing infrastructure. This research was funded by PhD grants of the Institute for the Promotion of Innovation through Science and Technology in Flanders (IWT-Vlaanderen) to B.V.d.P. and I.B. FWO-Vlaanderen is acknowledged for providing an International Mobility grant to B.V.d.P., a doctoral grant to T.N. and a post-doctoral fellowship to S.S.

## References

[B1] Giovannoni JJ Genetic regulation of fruit development and ripening Plant Cell 2004 16 S170 S180 10.1105/tpc.019158 15010516 PMC2643394

[B2] Hamilton AJ Bouzayen M Grierson D Identification of a tomato gene for the ethylene-forming enzyme by expression in yeast Proc Natl Acad Sci USA 1991 88 7434 7437 10.1073/pnas.88.16.7434 1714605 PMC52310

[B3] Dong JG Fernandezmaculet JC Yang SF Purification and characterization of 1-aminocyclopropane-1-carboxylate oxidase from apple fruit Proc Natl Acad Sci USA 1992 89 9789 9793 10.1073/pnas.89.20.9789 1409700 PMC50218

[B4] Hoffman NE Yang SF Mckeon T Identification of 1-(malonylamino)cyclopropane-1-carboxylic acid as a major conjugate of 1-aminocyclopropane-1-carboxylic acid, an ethylene precursor in higher-plants Biochem Biophys Res Commun 1982 104 765 770 10.1016/0006-291X(82)90703-3 7073714

[B5] Liu Y Hoffman NE Yang SF Relationship between the malonylation of 1-aminocyclopropane-1-carboxylic acid and D-amino acids in mung-bean hypocotyls Planta 1983 158 437 441 10.1007/BF00397737 24264853

[B6] Martin MN Cohen JD Saftner RA A New 1-aminocyclopropane-1-carboxylic acid-conjugating activity in tomato fruit Plant Physiol 1995 109 917 926 10.1104/pp.109.3.917 8552720 PMC161393

[B7] Staswick PE Tiryaki I The oxylipin signal jasmonic acid is activated by an enzyme that conjugates it to isoleucine in Arabidopsis Plant Cell 2004 16 2117 2127 10.1105/tpc.104.023549 15258265 PMC519202

[B8] Boller T Herner RC Kende H Assay for and enzymatic formation of an ethylene precursor, 1-aminocyclopropane-1-carboxylic acid Planta 1979 145 293 303 10.1007/BF00454455 24317737

[B9] Matas AJ Yeats TH Buda GJ Zheng Y Chatterjee S Tohge T Ponnala L Adato A Aharoni A Stark R Tissue- and cell-type specific transcriptome profiling of expanding tomato fruit provides insights into metabolic and regulatory specialization and cuticle formation Plant Cell 2011 23 3893 3910 10.1105/tpc.111.091173 22045915 PMC3246317

[B10] Teyssier E Bernacchia G Maury S Kit AH Stammitti-Bert L Rolin D Gallusci P Tissue dependent variations of DNA methylation and endoreduplication levels during tomato fruit development and ripening Planta 2008 228 391 399 10.1007/s00425-008-0743-z 18488247

[B11] Neily MH Matsukura C Maucourt M Bernillon S Deborde C Moing A Yin YG Saito T Mori K Asamizu E Enhanced polyamine accumulation alters carotenoid metabolism at the transcriptional level in tomato fruit over-expressing spermidine synthase J Plant Physiol 2011 168 242 252 10.1016/j.jplph.2010.07.003 20708298

[B12] Centeno DC Osorio S Nunes-Nesi A Bertolo ALF Carneiro RT Araujo WL Steinhauser MC Michalska J Rohrmann J Geigenberger P Malate plays a crucial role in starch metabolism, ripening, and soluble solid content of tomato fruit and affects postharvest softening Plant Cell 2011 23 162 184 10.1105/tpc.109.072231 21239646 PMC3051241

[B13] Brown MM Hall JL Ho LC Sugar uptake by protoplasts isolated from tomato fruit tissues during various stages of fruit growth Physiol Plant 1997 101 533 539 10.1111/j.1399-3054.1997.tb01034.x

[B14] Cheng YC Wang TT Chen JH Lin TT Spatial-temporal analyses of lycopene and sugar contents in tomatoes during ripening using chemical shift imaging Postharvest Biol Technol 2011 62 17 25 10.1016/j.postharvbio.2011.04.006

[B15] Luengwilai K Beckles DM Structural investigations and morphology of tomato fruit starch J Agric Food Chem 2009 57 282 291 10.1021/jf802064w 19093869

[B16] Wang F Smith AG Brenner ML Temporal and spatial expression pattern of sucrose synthase during tomato fruit-development Plant Physiol 1994 104 535 540 12232103 10.1104/pp.104.2.535 PMC159228

[B17] Smillie RM Hetherington SE Davies WJ Photosynthetic activity of the calyx, green shoulder, pericarp, and locular parenchyma of tomato fruit J Exp Bot 1999 50 707 718

[B18] Lemaire-Chamley M Petit J Garcia V Just D Baldet P Germain V Fagard M Mouassite M Cheniclet C Rothan C Changes in transcriptional profiles are associated with early fruit tissue specialization in tomato Plant Physiol 2005 139 750 769 10.1104/pp.105.063719 16183847 PMC1255993

[B19] Mounet F Moing A Garcia V Petit J Maucourt M Deborde C Bernillon S Le Gall G Colquhoun I Defernez M Gene and metabolite regulatory network analysis of early developing fruit tissues highlights New candidate genes for the control of tomato fruit composition and development Plant Physiol 2009 149 1505 1528 10.1104/pp.108.133967 19144766 PMC2649409

[B20] Moco S Capanoglu E Tikunov Y Bino RJ Boyacioglu D Hall RD Vervoort J De Vos RCH Tissue specialization at the metabolite level is perceived during the development of tomato fruit J Exp Bot 2007 58 4131 4146 10.1093/jxb/erm271 18065765

[B21] Brecht JK Locular Gel Formation in Developing Tomato Fruit and the Initiation of Ethylene Production Hortscience 1987 22 476 479

[B22] Van de Poel B Bulens I Hertog MLAT Van Gastel L De Proft MP Nicolai BM Geeraerd AH Model-based classification of tomato fruit development and ripening related to physiological maturity Postharvest Biol Technol 2012 67 59 67

[B23] Atta-Aly MA Brecht JK Huber DJ Ripening of tomato fruit locule gel tissue in response to ethylene Postharvest Biol Technol 2000 19 239 244 10.1016/S0925-5214(00)00099-5

[B24] Martin MN Saftner RA Purification and characterization of 1-aminocyclopropane-1-carboxylic acid N-malonyltransferase from tomato fruit Plant Physiol 1995 108 1241 1249 12228541 10.1104/pp.108.3.1241 PMC157479

[B25] Van de Poel B Bulens I Markoula A Hertog MLAT Deesen R Wirtz M Vandoninck S Oppermann Y Keulemans J Hell R Targeted systems biology profiling of tomato fruit reveals coordination of the yang cycle and a distinct regulation of ethylene biosynthesis during postclimacteric ripening Plant Physiol 2012 160 Markoula A 1498 1514 22977280 10.1104/pp.112.206086 PMC3490579

[B26] Yang SF Hoffman NE Ethylene biosynthesis and its regulation in higher-plants Annu Rev Plant Physiol Plant Mol Biol 1984 35 155 189 10.1146/annurev.pp.35.060184.001103

[B27] Lincoln JE Cordes S Read E Fischer RL Regulation of gene-expression by ethylene during lycopersicon-esculentum (tomato) fruit-development Proc Natl Acad Sci U S A 1987 84 2793 2797 10.1073/pnas.84.9.2793 3472237 PMC304745

[B28] Lincoln JE Fischer RL Diverse mechanisms for the regulation of ethylene-inducible gene-expression Mol Gen Genet 1988 212 71 75 10.1007/BF00322446 3163768

[B29] Penarrubia L Aguilar M Margossian L Fischer RL An antisense gene stimulates ethylene hormone production during tomato fruit ripening Plant Cell 1992 4 681 687 12297659 10.1105/tpc.4.6.681 PMC160164

[B30] Kneissl ML Deikman J The tomato E8 gene influences ethylene biosynthesis in fruit but not in flowers Plant Physiol 1996 112 537 547 12226407 10.1104/pp.112.2.537 PMC157976

[B31] Prescott AG A dilemma of dioxygenases (or where biochemistry and molecular-biology fail to meet) J Exp Bot 1993 44 849 861 10.1093/jxb/44.5.849

[B32] Pickard WF The role of cytoplasmic streaming in symplastic transport Plant Cell Environ 2003 26 1 15 10.1046/j.1365-3040.2003.00845.x

[B33] Lucas WJ Lee JY Plant cell biology - plasmodesmata as a supracellular control network in plants Nat Rev Mol Cell Biol 2004 5 712 726 10.1038/nrm1470 15340379

[B34] Chen XY Kim JY Transport of macromolecules through plasmodesmata and the phloem Physiol Plant 2006 126 560 571 10.1111/j.1399-3054.2006.00630.x

[B35] Oparka KJ Cruz SS The great escape: phloem transport and unloading of macromolecules Annu Rev Plant Physiol Plant Mol Biol 2000 51 323 347 10.1146/annurev.arplant.51.1.323 15012195

[B36] De Boer AH Volkov V Logistics of water and salt transport through the plant: structure and functioning of the xylem Plant Cell Environ 2003 26 87 101 10.1046/j.1365-3040.2003.00930.x

[B37] Bradford KJ Yang SF Xylem transport of 1-aminocyclopropane-1-carboxylic acid, an ethylene precursor, in waterlogged tomato plants Plant Physiol 1980 65 322 326 10.1104/pp.65.2.322 16661182 PMC440319

[B38] Apelbaum A Yang SF Biosynthesis of stress ethylene induced by water deficit Plant Physiol 1981 68 594 596 10.1104/pp.68.3.594 16661963 PMC425945

[B39] Albacete A Ghanem ME Martinez-Andujar C Acosta M Sanchez-Bravo J Martinez V Lutts S Dodd IC Perez-Alfocea F Hormonal changes in relation to biomass partitioning and shoot growth impairment in salinized tomato (Solanum lycopersicum L.) plants Journal of Experimental Botany 2008 59 4119 4131 10.1093/jxb/ern251 19036841 PMC2639025

[B40] Morris DA Larcombe NJ Phloem transport and conjugation of foliar-applied 1-aminocyclopropane-1-carboxylic acid in cotton (gossypium-hirsutum L) J Plant Physiol 1995 146 429 436 10.1016/S0176-1617(11)82004-3

[B41] Tophof S Martinoia E Kaiser G Hartung W Amrhein N Compartmentation and transport of 1-aminocyclopropane-1-carboxylic acid and N-malonyl-1-aminocyclopropane-1-carboxylic acid in barley and wheat mesophyll-cells and protoplasts Physiol Plant 1989 75 333 339 10.1111/j.1399-3054.1989.tb04635.x

[B42] Saftner RA Martin MN Transport of 1-aminocyclopropane-1-carboxylic acid into isolated maize mesophyll vacuoles Physiol Plant 1993 87 535 543 10.1111/j.1399-3054.1993.tb02504.x

[B43] Jiao XZ Philosophhadas S Su LY Yang SF The conversion of 1-(malonylamino)cyclopropane-1-carboxylic acid to 1-aminocyclopropane-1-carboxylic acid in plant-tissues Plant Physiol 1986 81 637 641 10.1104/pp.81.2.637 16664869 PMC1075390

[B44] Hanley KM Meir S Bramlage WJ Activity of aging carnation flower parts and the effects of 1-(malonylamino)cyclopropane-1-carboxylic acid-induced ethylene Plant Physiol 1989 91 1126 1130 10.1104/pp.91.3.1126 16667122 PMC1062129

[B45] Bouzayen M Latche A Alibert G Pech JC Intracellular sites of synthesis and storage of 1-(malonylamino)cyclopropane-1-carboxylic acid in acer-pseudoplatanus cells Plant Physiol 1988 88 613 617 10.1104/pp.88.3.613 16666357 PMC1055633

[B46] Bouzayen M Latche A Pech JC Marigo G Carrier-mediated uptake of 1-(malonylamino)cyclopropane-1-carboxylic acid in vacuoles isolated from catharanthus-roseus cells Plant Physiol 1989 91 1317 1322 10.1104/pp.91.4.1317 16667182 PMC1062185

[B47] Roje S S-adenosyl-L-methionine: beyond the universal methyl group donor Phytochemistry 2006 67 1686 1698 10.1016/j.phytochem.2006.04.019 16766004

[B48] Van de Poel B Bulens I Oppermann Y Hertog MLAT Nicolai BM Sauter M Geeraerd AH S-adenosyl-l-methionine usage during climacteric ripening of tomato in relation to ethylene and polyamine biosynthesis and transmethylation capacity Physiol Plantarium 2013 148 176 188 10.1111/j.1399-3054.2012.01703.x 23020643

[B49] Palmieri L Arrigoni R Blanco E Carrari F Zanor MI Studart-Guimaraes C Fernie AR Palmieri F Molecular identification of an Arabidopsis S-adenosylmethionine transporter. Analysis of organ distribution, bacterial expression, reconstitution into liposomes, and functional characterization Plant Physiol 2006 142 855 865 10.1104/pp.106.086975 16950860 PMC1630753

[B50] Bulens I Van de Poel B Hertog MLAT De Proft MP Geeraerd AH Nicolai BM Protocol: an updated integrated methodology for analysis of metabolites and enzyme activities of ethylene biosynthesis Plant Methods 2011 7 17 **(**doi:10.1186/1746-4811-7-17) 10.1186/1746-4811-7-17 21696643 PMC3142538

[B51] Van de Poel B Bulens I Lagrain P Pollet J Hertog MLAT Lammertyn J De Proft MP Nicolai BM Geeraerd AH Determination of S-adenosyl-L-methionine in fruits by capillary electrophoresis Phytochem Anal 2010 21 602 608 10.1002/pca.1241 20690158

[B52] Bradford MM Rapid and sensitive method for quantitation of microgram quantities of protein utilizing principle of protein-Dye binding Anal Biochem 1976 72 248 254 10.1016/0003-2697(76)90527-3 942051

[B53] D’Hertog W Overbergh L Lage K Ferreira GB Maris M Gysemans C Flamez D Cardozo AK Van den Bergh G Schoofs L Proteomics analysis of cytokine-induced dysfunction and death in insulin-producing INS-1E cells Mol Cell Proteomics 2007 6 2180 2199 10.1074/mcp.M700085-MCP200 17921177

[B54] Schneider CA Rasband WS Eliceiri KW NIH image to ImageJ: 25 years of image analysis Nature Methods 2012 9 671 675 10.1038/nmeth.2089 22930834 PMC5554542

