# Supplementary material for: Tissue specific analysis reveals a differential organization and regulation of both ethylene biosynthesis and E8 during climacteric ripening of tomato
Source: BMC Plant Biol. 2014 Jan 8;14:11. doi: 10.1186/1471-2229-14-11 (PMC3900696; doi:10.1186/1471-2229-14-11)
Supplement: Additional file 1: Figure S1 — Additional Western blots to characterize the two bands. Figure S2. MALDI-TOF/TOF peptide analysis the two bands. Figure S3. Coomassie stained SDS-PAGE of the purified His-tagged ACO and E8 proteins. Figure S4. Identification of the purified ACO and E8 after overexpression. Figure S5. Sequence properties of the custom polyclonal anti-ACO antibody. Figure S6. Sequence alignment between tomato ACO1 and E8. [file 1471-2229-14-11-S1.doc]

Additional Material

**Tissue specific analysis reveals a differential organization and regulation of both ethylene biosynthesis and E8 during climacteric ripening of tomato**

Bram Van de Poel, Nick Vandenzavel, Cindy Smet, Toon Nicolay, Inge Bulens, Ifigeneia Mellidou, Sandy Vandoninck, Maarten L.A.T.M. Hertog, Rita Derua, Stijn Spaepen, Jos Vanderleyden, Etienne Waelkens, Maurice P. De Proft, Bart M. Nicolai & Annemie H. Geeraerd

**Overview additional figures**

Additional Figure S1. Additional Western blots to characterize the two bands.

Additional Figure S2. MALDI-TOF/TOF peptide analysis the two bands.

Additional Figure S3. Coomassie stained SDS-PAGE of the purified His-tagged ACO and E8 proteins.

Additional Figure S4. Identification of the purified ACO and E8 after overexpression.

Additional Figure S5. Sequence properties of the custom polyclonal anti-ACO antibody.

Additional Figure S6. Sequence alignment between tomato ACO1 and E8.


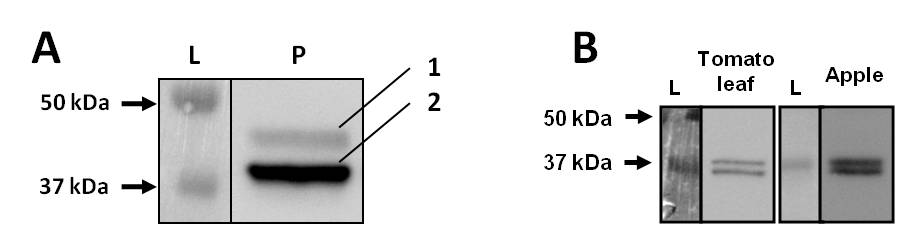


Additional Figure S1. Additional Western blots to characterize the two bands. (A) Western blot with commercial anti-ACO antibodies (sc-12781, Santa Cruz Biotechnologies, Inc., Santa Cruz, CA, USA) of pink (P) tomato fruit extracts identifying two bands (1 and 2). (B) Western blots confirming the presence of the two bands in tomato leaf tissue and apple fruit with the custom anti-ACO antibody. L: ladder.


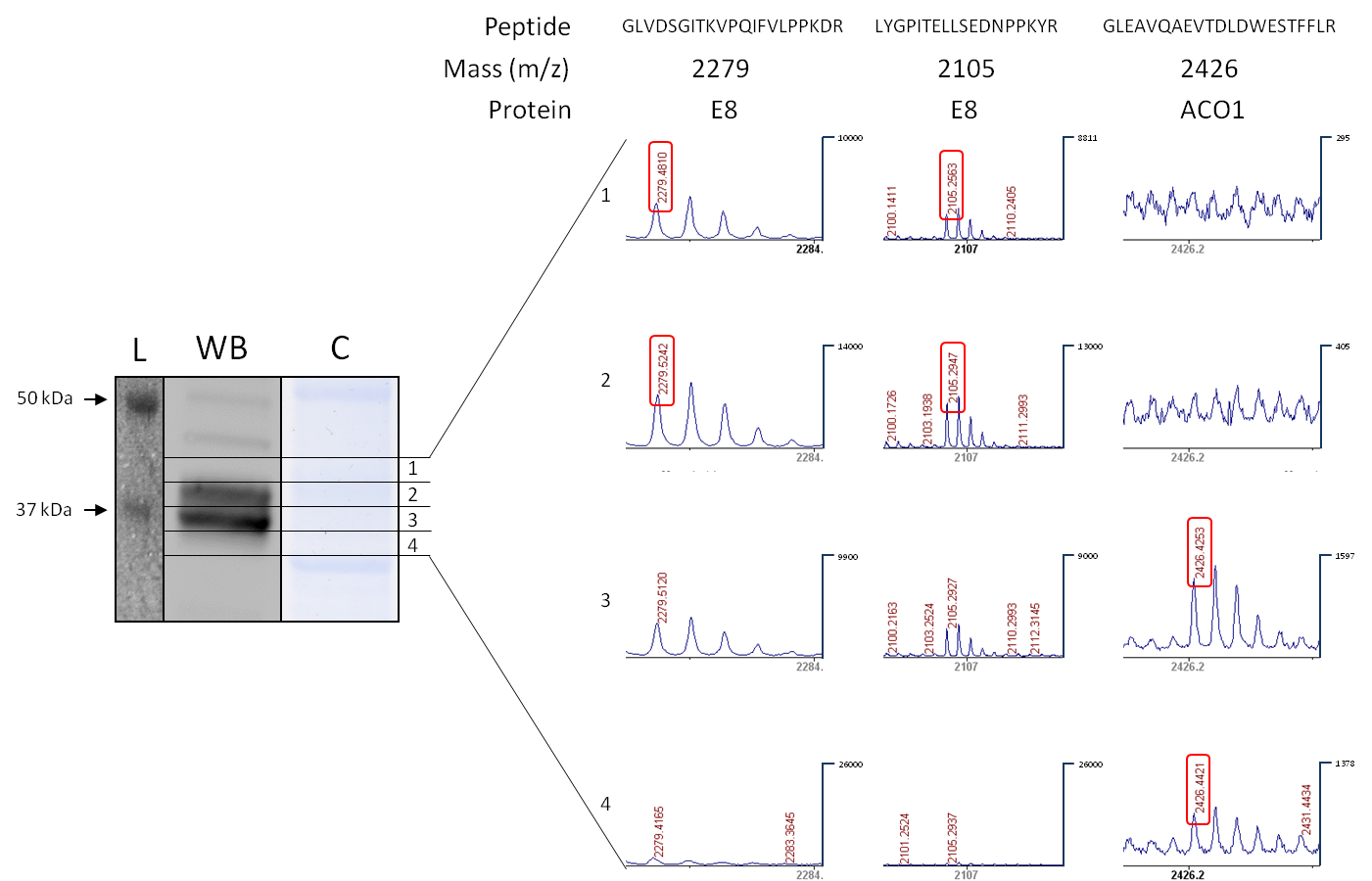


Additional Figure S2. MALDI-TOF/TOF peptide analysis of cut SDS-PAGE samples after trypsin digestion. Four band of a coomassie stained gel (C) were cut out based upon the Western blot (WB) results: (1) just above the upper band, (2) the upper band, (3) the lower band and (4) just below the lower band. Three unique and specific peptides were observed in a gradient along the 4 cut bands. The E8 peptides (GLVDSGITKVPQIFVLPPKDR and LYGPITELLSEDNPPKYR) associated with the upper band (E8 = 41 kDa) and the ACO1 peptide (GLEAVQAEVTDLDWESTFFLR) associated with the lower band (ACO1 = 36 kDa). L: ladder. x-axis: mass (m/z); y-axis: % intensity.


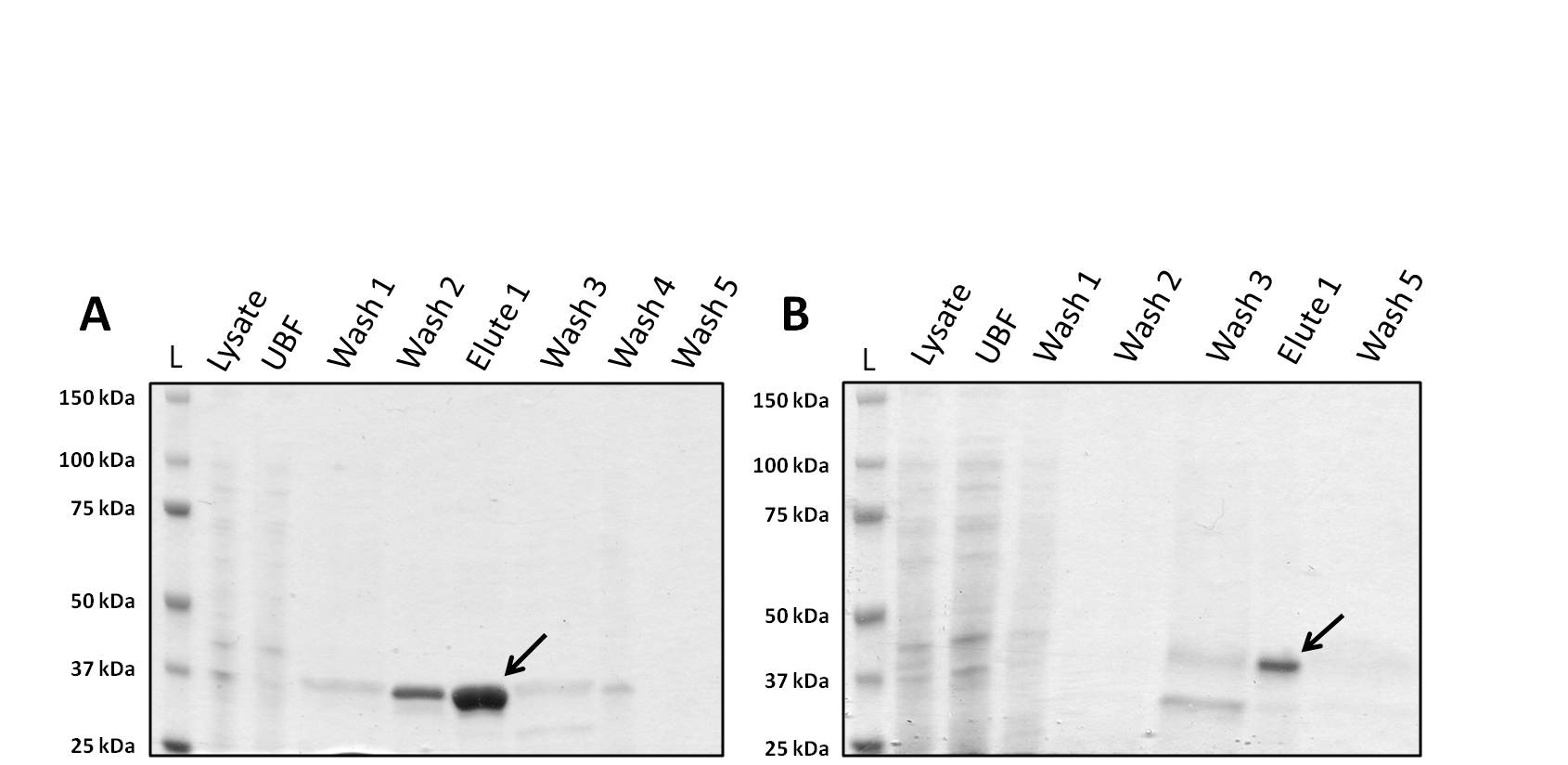


Additional Figure S3. Coomassie stained SDS-PAGE of the purified HIS tagged ACO (A) and E8 (B) proteins. UBF represents the unbound fractions. The different wash steps contain different concentrations of imidazole. Wash 1: 30 mM imidazole; Wash 2: 50 mM imidazole; Wash 3: 80 mM imidazole; Wash 4: 250 mM imidazole; Wash 5: 500 mM imidazole. The purified proteins were eluted with 80 mM imidazole (Elute 1) and are indicated with a black arrow. L: ladder.


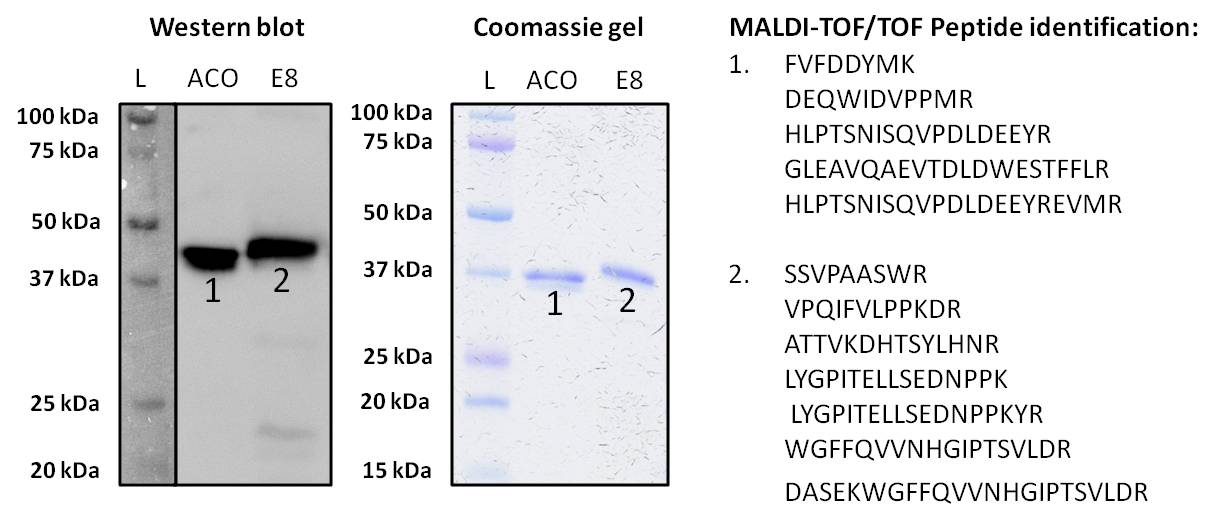


Additional Figure S4. Identification of the purified ACO and E8 after overexpression by Western blotting (custom anti-ACO antibodies) and MALDI-TOF/TOF analysis after SDS-PAGE and coomassie staining. Different unique peptides were found after trypsin digestion representing ACO1 (1) and E8 (2). L: ladder.

**A**

170 180 190 200 210 220 230

|...|....| ....|....| ....|....| ....|....| ....|....| ....|....|

ACO1 LIKGLRAHTD AGGIILLFQD DKVSGLQLLK DEQWIDVPPM RHSIVVNLGD QLEVITNGKY

AB ---------- --------QD DKVSGLQLLK DE-------- ---------- ----------

** ********** **

**B**

230 240 250 260 270 280 290

|....|....| ....|....| ....|....| ....|....| ....|....| ....|....| E8 TMGTIQHTDIG FVTILLQDDM GGLQVLHQNH WVDVPPTPGS LVVNIGDFLQ LLSNDKYLSV

AB ----------- ------QDDK ---------- ------VSG- --------LQ LLKDE-----

*** ..* ** **.::

Additional Figure S5. Sequence properties of the custom polyclonal anti-ACO antibody. (A) Partial protein sequence alignment of ACO1 (P05116) and the custom polyclonal anti-ACO antibody epitope (AB) showing complete homology. (B) Partial protein sequence alignment of E8 (P10967) and the custom polyclonal anti-ACO antibody epitope (AB) showing possible sites of interaction of the polyclonal antibody with E8.

ACO1 ------------------------------------------------------------

E8 MESPRVEESYDKMSELKAFDDTKAGVKGLVDSGITKVPQIFVLPPKDRAKKCETHFVFPV

ACO1 ----------------MEMIKDACENWGFFELVNHGIPHEVMDTVEKMTKGHYKKCME--

E8 IDLQGIDEDPIKHKEIVDKVRDASEKWGFFQVVNHGIPTSVLDRTLQGTRQFFEQDNEVK

:: ::**.*:****::****** .*:* . : *: .::: *

ACO1 ------QRFKELVASKGLEAVQAEVTDLDWESTFFLRHLPTSNISQVPDLDEEYREVMRD

E8 KQYYTRDTAKKVVYTSNLDLYKSSVPAASWRDTIFCYMAPNP--PSLQEFPTPCGESLID

: *::* :. *: ::.* .*..*:* *. .: :: * : *

ACO1 FAKRLEKLAEELLDLLCENLGLEKGYLKNAFYGSKGPNFGTKVSNYPPCPKPDLIKGLRA

E8 FSKDVKKLGFTLLELLSEGLGLDRSYLKDYMDC---FHLFCSCNYYPPCPQPELTMGTIQ

*:* ::**. **:**.* ***::.***: : .: . . *****:*:* *

ACO1 HTDAGGIILLFQDDKVSGLQLLKDEQWIDVPPMRHSIVVNLGDQLEVITNGKYKSVLHRV

E8 HTDIGFVTILLQ-DDMGGLQVLHQNHWVDVPPTPGSLVVNIGDFLQLLSNDKYLSVEHRA

*** * : :*:* *.:.***:*::::*:**** *:***:** *::::* ** ** **.

ACO1 IAQTDGTRMSLASFYNPGSDAV--IYP-AKTLVEKEAEESTQVYPKFVFDDYMKL--YAG

E8 ISNNVGSRMSITCFFGESPYQSSKLYGPITELLS---EDNPPKYRATTVKDHTSYLHNRG

*::. *:***::.*: . :* . *:. *:. * ...*: . *

ACO1 LKFQAKEPRFEAMKAMESDPIASA

E8 LDGTSALSRYKI------------

*. : *::

Additional Figure S6. Sequence alignment between tomato ACO1 and E8 (Clustal-Omega; EMBL-EBI, online) showing only 34.31 % sequence homology.
